# Reduction of HIV-1 infectivity by exploiting the cellular ERAD machinery

**DOI:** 10.1186/1742-4690-10-S1-P11

**Published:** 2013-09-19

**Authors:** Antonio Casini, Michele Olivieri, Oscar Burrone, Anna Cereseto

**Affiliations:** 1 Centre for Integrative Biology (CIBIO), University of Trento, Mattarello, Italy; 2 International Centre for Genetic Engineering and Biotechnology (ICGEB), Trieste, Italy

## Background

During viral particle production HIV-1 exploits the endoplasmic reticulum (ER) to synthesize two factors: Vpu and gp160. These viral proteins contain a secretion signal and are thus inserted co-translationally in the ER. The gp160 glycoprotein, undergoes extensive post-translational modification during its trafficking through the ER. Notably, in these same compartments also operates the ER-associated degradation (ERAD) pathway, a quality control mechanism that promotes the proteasomal degradation of misfolded proteins that are trafficking through the secretory pathway.

Since the correct maturation of the gp160 precursor is absolutely essential to produce infectious viral particles, we reasoned that inducing the degradation of gp160 in producing cells may generate an impaired viral progeny.

## Materials and methods

We exploited a recently developed technique that allows the specific degradation of proteins trafficking within the secretory pathway [1], in this case the precursor gp160. This method is based on chimeric molecules named degradins, made of two functional moieties: a target recognition domain and a degradation-inducing domain. The latter corresponds to a truncated form of the ER-resident cellular protein SEL1L which plays a fundamental role in promoting the entrance into the ERAD pathway of misfolded proteins. In the present study we used as target recognition moieties different single-chain antibodies specific for gp120 and a portion of the luminal domain of CD4, which is known to bind gp160 in the ER.

We tested the efficacy of the different degradins both by co-transfection with gp160 in 293T cells and by measuring the reduction in the infectivity of vectors produced in degradin-expressing cells.

## Results

By co-expression of gp160 in 293T cells with different gp160-specific degradins (1339-SEL1L, VRC01-SEL1L, VRC03-SEL1L, CD4-SEL1L) we obtained decreased cellular levels of gp160. This effect was specific, as demonstrated by insignificant modulation of gp160 levels by an irrelevant degradin (1C10-SEL1L), and no downregulation of irrelevant targets (Tetherin, human MHC-I) by gp160-specifk degradins. In addition, co-expression of the target recognition moieties contained in gp160-specific degradins fused to the KDEL ER retention signal, was unable to lower the intracellular levels of the viral target.

Finally, we prove that the expression of anti-gp160 degradins in producing cells strongly impairs viral infectivity.

## Conclusions

This study proposes a new strategy to reduce HIV-1 infectivity by interfering with the correct formation of viral particles. Former approaches obtained reduction of gp160 incorporation during viral particle assembly through retention of the gp160 precursor in the ER. Conversely, the strategy developed in this study is based on the induction of the active degradation of gp160 through the ERAD pathway during gp160 transition through the ER.
